# Deliberate exposure of humans to chlorine-the aftermath of Ebola in West Africa

**DOI:** 10.1186/s13756-016-0144-1

**Published:** 2016-11-14

**Authors:** Shaheen Mehtar, Andre N. H. Bulabula, Haurace Nyandemoh, Steve Jambawai

**Affiliations:** 1 Infection Control Africa Network, Cape Town, South Africa; 2 Unit for IPC, H9 Floor, Tygerberg Hospital, Faculty of Medicine and Health Sciences, Stellenbosch University, Francie van Zijl Drive, Cape Town, South Africa; 3 Ministry of Health & Sanitation, Freetown, Sierra Leone

**Keywords:** Chlorine, Spray, Deliberate exposure, Humans, Healthcare workers, West Africa, Ebola, Infection control, Decontamination, Adverse events, Side effects, Occupational exposure

## Abstract

**Background:**

During the recent Ebola outbreak, spraying of the environment and humans, including healthcare workers, with chlorine was wide spread in affected African countries; adverse clinical effects are reported here.

**Methods:**

A cross sectional survey by interview of 1550 volunteers consisting of 500 healthcare workers (HCW), 550 Ebola survivors (EVD) and 500 quarantined asymptomatic Ebola contacts (NEVD) was conducted. Demographics, frequency of exposure to chlorine, clinical condition after chlorine exposure particularly eye, respiratory and skin conditions were noted. The length of time HCWs worked in Ebola Treatment Units (ETU), and use of personal protective equipment was recorded. Verbal consent was obtained from all participants and all responses remained anonymous. Permission and assistance from the guardian or parent was sought for those below 18 years of age.

**Results:**

493/500 HCW, 550/550 EVD and 477/500 NEVD were sprayed at least once with 0 · 5 % chlorine. Following even a single exposure, an increase in the number of eye (all three groups) and respiratory symptoms (in HCW & EVD) was reported (*p* < 0 · 001); after multiple exposure, respiratory and skin symptoms increased. In HCW, multiple vs single exposure was associated with an increase in respiratory (OR = 32 (95 % CI 22 –49) *p* < 0.001), eyes (OR = 30 (95 % CI 21 –43) *p* < 0.001) and skin conditions (OR = 22 (95 % CI 15–32) *p* < 0.001). The available personal protective equipment neither reduced nor prevented the adverse effects of chlorine.

**Conclusion:**

Reported exposure to chlorine has usually been accidental. Despite the lack of evidence as a recognised outbreak control measure, deliberate exposure of humans to chlorine spray was wide spread in Africa during the Ebola epidemic resulting in serious detrimental health effects on humans. We strongly recommend that this practice be banned and that alternative safer methods be used.

## Background

The 2014–2015 Ebola outbreak had a devastating impact on Africa. The worst hit countries, Liberia, Sierra Leone and Guinea reported more than 28646 cases of Ebola and over 11,323 deaths [1]. Viral haemorrhagic fever outbreaks are endemic in rural areas of West and East Africa, and Ebola in particular reflects a close relationship between human and non-human primates, living in these forests [2, 3]. By the time the outbreak was officially recognised, many front line healthcare workers (HCW), both from national and international organisations managing clinical cases of Ebola acquired the disease. Of the 852 HCW who were diagnosed with Ebola, 492 (58 %) died [1]. While HCW did their best to contain the spread of Ebola, the lack of appropriate training in infection prevention and control (IPC) and inadequate resources led to a high mortality rate [4]. Infection prevention and control (IPC) guidance from various organisations such as Centers for Disease Control [5, 6] Médecins Sans Frontières [7]_,_ World Health Organisation [8] amongst others, were rapidly deployed and these documents were mainly based on experience in other types of outbreaks such as cholera. The practice of extensive spraying of 0.5 % chlorine on both animate and inanimate objects, including humans who were either in direct, or indirect, contact with Ebola [6, 7] was used despite WHO recommendations to the contrary [8]. The IPC (Ebola) guidelines for West Africa contained instructions to make up chlorine from powder or liquid at concentrations of 0 · 5 % (5000 ppm) (for all surface disinfection including human clothing) and 0 · 05 % (500 ppm) chlorine for cleaning hands (http://www.cdc.gov/vhf/ebola/hcp/mixing-chlorine-solutions.html [9] and was part of the 3-day training for healthcare workers preparing to work in Ebola affected countries (http://www.cdc.gov/vhf/ebola/hcp/safety-training-course/training-toolkit.html) [10].

Chlorine based solutions have been widely used as effective broad spectrum disinfectants for many years and are widely available for both commercial and domestic use; it is among the ten highest volume chemicals manufactured in the United States and is generally produced under strictly controlled conditions. Chlorine is a noxious substance especially in its gaseous form [11]. The symptoms of acute chlorine poisoning are well documented but mainly from accidental exposure reports [12–14], which require immediate medical attention. The main symptoms are tightness of the chest [15], respiratory distress, visual disturbance, loss of visual acuity [13]. Skin reactions such as burning and dermatitis are noted following both acute and chronic exposure to chlorine [5, 9],.

This study documents the adverse health effects resulting from deliberate chlorine spraying of humans (particularly HCWs) during the Ebola outbreak in Sierra Leone.

## Methods

A cross sectional survey was conducted between August and November 2015 (4 months) interviewing 1550 volunteers. The objective was to determine the adverse effects following known exposure to chlorine spray on humans, with an aim to document prevalence of eye, chest and skin conditions amongst the volunteers who were interviewed and to determine whether chlorine was a risk factor in the occurrence of these clinical conditions.

Definitions used in this paper are as follows:

- Eye condition: defined as any sign or symptom (pain, worse sight than before, painful eyes) that occurred during the Ebola epidemic after exposure to chlorine spray. Specific treatment and/or a visit to the eye clinic were noted.
- Chest condition: Participants presenting with cough (violent or not, producing sputum or not), burning throat, chest tightness or difficulty in breathing, after exposure to chlorine.
- Skin condition: was defined as skin irritation, dermatitis, cracking or burning sensation after being exposed to chlorine.
- Health care worker (HCW): included doctors, nurses, lab technicians, community healthcare staff and hygienists working in the ETUs and with patients both in the healthcare facilities and in the community.
- EVD Survivors (EVD): were those who were declared Ebola free after recovering from the clinical disease and usually confirmed as being Ebola antibody negative.
- Non-EVD persons (NEVD): were those who did not have clinical Ebola but were contacts of known or suspected EVD cases and were under quarantine in their homes.

The healthcare workers were actively involved in the direct clinical care of Ebola cases. The hygienist cleaned the healthcare facility, removed waste from the Red Zone which was the part of the ETU were clinical cases of Ebola were cared for. The other major job was spraying other HCWs as they emerged from the Red Zone and often with minimal protection.

The inclusion criteria were HCW, EVD and their contacts who were quarantined (NEVD) and had been directly sprayed with chlorine. Those who had no history of direct chlorine spray exposure were excluded although it was noted that some participants such as HCW and NEVD were present when the surrounding environment was sprayed because it was impossible to get away from a total chlorine free environment. History of smoking was recorded. No confounding factor was included in the analysis, however, Ebola disease itself is known to cause damage to the eyes and this was borne in mind.

One thousand five hundred and fifty volunteers were recruited from one of the following three groups: Healthcare workers (HCW) who were dealing with clinical cases of Ebola (*n* = 500); survivors of either confirmed or suspected cases of clinical Ebola (EVD) (*n* = 550) and finally those Ebola contacts with no clinical symptoms at the time of interview but had been exposed to suspected or confirmed cases of Ebola (NEVD) (*n* = 500) and were under quarantine.

Two qualified nurses conducted interviews between August and November 2015 (4 months). The participants were visited by one of the two interviewers, either at the place of work (HCW), or at their residence (EVD and NEVD groups) in the community They visited the participants in rural, semi-urban and urban areas one (SJ) worked with Ebola survivors (EVD) while the other (HN) interviewed HCW and NEVD groups. A simple yet exact questionnaire was devised which required either a “yes/no” or “don’t know” answer; there were neither open ended answers nor narrative responses. The questionnaire was validated by a pilot study of 37 volunteers during an IPC training session in Sierra Leone in April 2015 where one of the interviewers was present. After minor amendments, the questionnaire was sent to the two interviewers to start collecting data.

The questionnaire covered demographics, the number of exposures to chlorine spray, when and where they were sprayed, and their state of health prior to being sprayed. Of particular interest were eye symptoms such as uveitis and loss of visual acuity which also constituted part of clinical picture of Ebola, therefore the presence of these symptoms prior to exposure were carefully recorded to establish that which was present prior to being sprayed to try and differentiate between the two risk factors- (one of the authors (SJ) was a qualified ophthalmic nurse practitioner). The replies were subjective but the change in visual acuity was evaluated by clinical examination.

Respiratory symptoms included tightness of the chest, shortness of breath, irritation of the throat and coughing, and skin conditions consisted of burning, cracking of the skin, and dermatitis. For HCWs, the length of time they worked in Ebola treatment units (ETU), frequency and use of personal protective equipment was recorded. Verbal consent was obtained from all voluntary participants, and the responses remained anonymous.

The demographic data was collected in larger age groups according to where exposure would most likely occur, for example either at the workplace or in the community. Thus, unusually, the sample was divided into age categories which were under the age of 18 years (minors) mainly EVD survivors and the NEVD group but not HCW; 19–35 years who constituted the younger adult including HCWs and from the other two categories; 36–50 years as the older workforce including HCWs, and over 50 years of age of whom fewer were employed as HCWs. For those under the age of 18 years, permission and assistance from the guardian or parent to complete the questionnaire was sought. During the interview participants were asked to recall their experience before and after exposure to chlorine spray, the number of times they were exposed and adverse effects if any, they encountered after being sprayed.

The group who had not been sprayed were also interviewed and their health condition was recorded however those that had not been sprayed were excluded from the final analysis.

The results were entered onto an Excel database and analysed (AB) using Stata version 13 statistical package. The forms remained in Sierra Leone and were available at SM’s next visit.

The clinical syndromes- eyes, chest and skin conditions, were dependent variables (binary). Demographics and the frequency of spraying with chlorine represented independent variables. Pearson Chi [2] was used to compare proportions. Univariate logistic regression analyses were performed to determine the relationship between the dependent and the independent variables, however multivariate analysis could not be performed. The results of the logistic regression were presented as odds ratio with their 95 % confidence intervals. The use of PPE (HCW group) and the history of Ebola disease (EVD group) were potential confounders.

Permission to conduct the study was obtained from the Ministry of Health and Sanitation, Sierra Leone.

## Results

A total of 1550 participants volunteered to be interviewed of whom there were 550 Ebola survivors (EVD), while HCW and NEVD community members made up 500 each; their recall of chlorine spray exposure was vivid as some had been exposed quite recently and on multiple occasions.

Of the 500 HCWs, nine were EVD survivors; one reported becoming blind and another as partially blind based upon clinical diagnosis from the eye clinic which they were still attending. It was unclear whether this was due to Ebola clinical disease or the chlorine spray but most likely the former. They were included in the final analysis because they had been sprayed. All HCWs were working in the ETUs in the Red Zone where according to the existing guidance, spraying of individuals and the environment was carried out constantly on a daily basis. Seven HCWs, (nurses =5, hygienist = 1, pharmacist =1) had not been sprayed directly and were excluded from the analysis, leaving 493 HCW questionnaires to be analysed. The clinical symptoms amongst these seven HCW were minimal; one reported an unrelated previous eye condition, another reported breathing difficulty plus a tight chest as well as dermatitis; and a nurse reported dermatitis. It is noteworthy that although not sprayed directly, all seven of the HCWs worked in ETUs where exposure to chlorine was a daily occurrence.

In the NEVD group, 23 persons were not sprayed with chlorine directly; 14 were under the age of 7 years and nine were between the ages of 22 and 30 years. There were 477/500 (95.4 %) who had direct chlorine spray exposure. Of the 23 NEVD who had not been exposed to chlorine spray, none of them reported any of the clinical conditions on the questionnaire. In the EVD group all 550 had been sprayed and were included in the final analysis.

### Demographics

The age distribution of the three groups that were exposed to chlorine spray is shown in Table 1 and were grouped into larger categories for convenience as described above. The dominant age distribution in the three groups was between 19 and 35 years. In the EVD survivor group 29 % where below the age of 18 years and 93 % were over 50 years of age. For the NEVD group, there were less than 3 % under the age of 18 years and <1 % over 50 years. Approximately 99 % of HCW were between the ages of 19–50 years. The majority of the participants resided in rural districts. The gender distribution is shown in Table 1 and reflects a predominance of males in the EVD and HCW groups and females in the NEVD group.

**Table 1:** Distribution of age, gender and residential areas for the three interview groups

Age (years)	EVD N (550) *n* (%)	Non EVD N (477) *n* (%)	HCW N (493) *n* (%)	*P*-value
<1 year	4 (0 · 7)	1 (0 · 2)		<0.001
2–18	155 (28 · 2)	12 (2 · 5)		
19–35	236 (42 · 9)	451 (94 · 5)	371 (75 · 4)	
36–50	104 (18 · 9)	10 (2 · 1)	118 (23 · 8)	
50+	51 (9 · 3)	3 (0 · 6)	4 (0 · 8)	
*Male*	*225 (50)*	*78 (16 · 35)*	*273 (55 · 6)*	*<0.001*
*Smoking*	*201 (36 · 5)*	*35 (73)*	*38 (7 · 7)*	*<0.001*
*Urban*	*50 (9)*	*190 (40)*	*74 (15)*	

### Exposure to chlorine

The majority of HCWs interviewed were either hygienists 231/493 (46.8 %) or nurses 224/493 (46 %); others were community health officers (*n* = 4), doctors, district social officers (*n* = 4), pharmacists (*n* = 6) and laboratory technician (*n* = 17). Sixty eight percent had worked in the ETUs for 4–6 months, and 28 % had been there for 7–8 months; 3 % were working in ETUs for 9–12 months. Single and multiple exposure was reported by 285/493 (58 %) in 208/493 (42 %) HCW respectively. EVD survivors were sprayed on a single occasion in 292/550 (53 %) and multiple exposure in 258/550 (47 %).

#### When and where sprayed

The recommended chlorine strength used for spraying was 0.5 % but there was no means of establishing the exact concentration of the mixture. No quantity was defined- spraying continued until the persons were totally soaked and all visible organic matter was washed away. This occurred each time there was any exposure to suspected or confirmed case of Ebola. Ninety two percent of the HCWs were sprayed when leaving the ETU, but they were also exposed when spraying others (23.5 %), or were present when others were being sprayed (23.6 %); some were sprayed in their own homes, or when visiting community dwellings (Table 2).

**Table 2:** Chlorine spraying in the three groups

Site	HCW		EVD		NEVD	
Total interviewed	500		550		500	
Not sprayed (excluded)	7		0		23	
Total analysed	*N* = 493	*%*	*N* = 550	*%*	*N* = 477	*%*
In own house (under quarantine)	9	*2*	0	*-*	440 0	*92*
Outside in the community	0	*-*	0	*-*	21	*4*
Pre transfer	0	*-*	162	*30*	15	*3*
Back of ambulance	61	*12*	547	*99*	38	*8 · 0*
Leaving ETU			550	*100*		
Red zone	455	*93*	120	*22*		
Spray others	113	*23*				
In room when spraying others	116	*24*				
EVD case house	16	*3*				
EVD suspect house	33	*7*				

*HCW* = healthcare workers; *EVD* = Ebola virus disease survivors; *NEVD* = non Ebola cases

Of the EVD survivors, 99.5 % reported being sprayed either prior to or during, transportation in the back of an ambulance with sealed windows and doors *en route* to the ETU- a trip that could last for 3 h in high temperatures. Those under quarantine (NEVD) were sprayed at home, but some were sprayed before transportation while others (8 %) were sprayed in the back of the ambulance during transportation (Table 2).

### Reported clinical adverse effects

Adverse events were reported by all three groups, albeit subjective, as defined in the methods section for eye, respiratory and skin conditions. Following a single chlorine exposure, significant increase in eye symptoms was reported in all three groups (*p* < 0.001), but the EVD survivors had the highest number of eye symptoms probably related to their clinical disease (Table 3). Respiratory symptoms were similar for EVD and HCW, and exceeded NEVD in significance (*p* < 0.001); skin irritation was highest amongst the HCW (33 · 6 %) (Table 3) and the use of 0.05 % chlorine used for washing hands could have contributed towards these symptoms.

**Table 3:** The clinical effects of single exposure amongst EVD, NEVD and HCW

Characteristic of single chlorine exposure	EVD (*N* = 292) n (%),	HCW (*N* = 285) n (%)	NEVD (*N* = 477) n (%)	*P*-value
Eye sight problem before	55 (19)	19 (7)	38 (8)	<0 · 001
Eye sight problem now	185 (63)	95 (34)	55 (12)	<0 · 001
Coughing	107 (37)	107 (38)	39 (8)	<0 · 001
Cough producing sputum	76 (26)	43 (15)	17 (4)	<0 · 001
Difficulty in breathing	86 (30)	66 (23)	24 (5)	<0 · 001
Chest tightness	100 (34)	109 (38)	30 (6)	<0 · 001
Burning throat	87 (30)	85 (30)	30 (6)	<0 · 001
Skin irritation	11 (4)	95 (34)	86 (18)	<0 · 001

Multiple exposure to chlorine was reported by EVD and HCW groups (Table 4). Pre-chlorine exposure eye symptoms were present in both EVD (19 · 4 %) and HCW (12 · 1 %) which increased to 64 and 59 · 1 % respectively post exposure, however this was not found to be statistically significant (*p* < 0.3). Following multiple exposure, respiratory tract symptoms such as difficulty in breathing, tightness in the chest and burning in the throat (*p* < 0 · 001) were more significant amongst HCWs (Table 4). Skin conditions were significantly higher in the HCW group, possibly due to extensive repeated exposure to chlorine (0.05 %) used for hand washing in the ETU.

**Table 4:** Multiple chlorine exposure effect on EVD and HCW compared

Characteristic of multiple exposure	EVD (*N* = 258) n (%),	HCW (*N* = 208) n (%)	*P*-value
Eye sight problem before	50 (19)	25 (12)	0 · 04
Eye sight problem now	165 (64)	123 (59)	0 · 3
Coughing	89 (35)	124 (60)	<0 · 001
Cough producing sputum	76 (30)	60 (29)	0 · 99
Difficulty in breathing	93 (36)	100 (48)	0 · 009
Chest tightness	92 (36)	131 (62 · 9)	<0 · 001
Burning throat	80 (31)	112 (54)	<0 · 001
Skin irritation	10 (4)	109 (52)	<0 · 001

For HCW, the clinical effect of multiple versus single exposure was documented. There was a significant increase in eye symptoms amongst those who had had multiple exposure increasing from 33 · 6 to 59 · 1 % (*p* < 0.001) and statistically significant increase in chest and skin conditions (Table 5). There was also an increase in the odds of chest condition (OR = 3.2 (95 % CI 2.0–4.9) *p* < 0.001), deterioration of their eyes (OR = 3.3 (95 % CI 2.2–5) *p* < 0.001) and skin condition (OR = 2.4 (95 % CI 1.6–3.6), *p* < 0.001) occurrence (unadjusted logistic regression) in the multiple exposure group. It was noteworthy that 57 · 5 % (184/320) of HCW with a chest condition also had an eye condition. There was no statistical significant difference between single and multiple exposure in the EVD group.

**Table 5:** Adverse events in HCW with single and multiple chlorine exposure compared

Characteristic	Single Cl_2_ exposure (*N* = 285) n(%),	Multiple Cl_2_ exposure (*N* = 208) n(%)	*P*-value
Eye sight problem before	19 (7)	25 (12)	0.04
Eye sight problem now	95 (34)	123 (59)	<0 · 001
Coughing	107 (38)	124 (60)	0 · 001
Cough producing sputum	43 (15)	60 (29)	<0 · 001
Difficulty in breathing	66 (23)	100 (48)	<0 · 001
Chest tightness	109 (38)	131 (63)	<0 · 001
Burning throat	85 (30)	112 (54)	<0 · 001
Skin irritation	95 (34)	109 (52)	<0 · 001

Always wearing eye protection during spraying was reported by 82 · 2 % (410/500), sometimes 14.6 % (73/500) or never 3.2 % (16/500), of HCWs. Skin protection (gloves and coverall) was worn 90 · 6, 9, and 0 · 4 % respectively for always, sometimes or never (Table 6). HCWs were more likely to wear skin protection, than eye protection (goggles or shields). Overall, 64 % of HCW reported chest conditions, and 48 % had eye problems following exposure. Despite the use of PPE, respiratory symptoms were reported by the nurses (*p* < 0.05) and skin conditions by the hygienists (*p* < 0.001) suggesting that wearing PPE did not protect HCW from adverse events of chlorine (Table 6).

**Table 6:** Category of staff and use of PPE and adverse events reported*DSO* district social officer, *CHO* community health officer

Job categories *n* = 293		Eye PPE		Skin PPE		Clinical symptoms		
Categories	n	Always	not always	always	not always	eye	chest	skin
Doctor	7	5	2	5	2			
Nurse	224	183	41	199	25	ns	0 · 05	ns
Hygienist	231	188	42	213	18	ns	ns	0 · 001
Pharmacist	6	5	1	5	1			
DSO	4	3	1	4	0			
CHO	4	4	0	4	0			
Lab technician	17	16	1	16	1			

### Limitations

The data was collected retrospectively towards the end of the EVD outbreak and the responses relied on memory of the participants. Most of them had vivid memories of being sprayed and the consequences. There was no shortage of volunteers to participate in the interviews and the sample could have been much larger, however the financial constraints limited the study to 1550 respondents. The study was conducted in the rural and semi urban districts of Sierra Leone and there was a limitation on travel and free movement at the time and so the interviewers were confined to their areas of residence and work.

The effect of Ebola on the eyes is well documented. In the EVD group it was difficult to establish the before and after adverse events attributable to chlorine alone. However, the NEVD and HCW who had not been infected provided a sizeable sample in this regard. Those presenting to the eye clinic for treatment provided a better insight into the clinical symptoms. Those that reported chest symptoms but these were absent at the time of interview, the interviewer recorded their replies. The presence of skin conditions were confounded by the use of 0.05 % chlorine for hand washing which the healthcare workers and community populations used frequently.

Finally, because the use of chlorine was widespread, it was difficult to find a large enough control sample size that had not been exposed to chlorine to be able to carry out a case- control study.

## Discussion

This Ebola outbreak was the largest recorded thus far in Africa and occurred outside the previously known range for the Ebola virus [1]. The rapid uncontrolled spread from rural to urban areas led to some highly unconventional containment practices, such as the widespread spraying of 0.5 % chlorine, which exposed many of the community and HCWs, to a noxious chemical substance. In the two separate CDC guidelines for dealing with Ebola, one for the West Africa (non- US healthcare settings) [6] http://www.cdc.gov/vhf/ebola/hcp/non-us-healthcare-settings.html and the other for the United States healthcare facilities [16] http://www.cdc.gov/vhf/ebola/healthcare-us/index.html recommendations on disinfection and the use of disinfectants were different. In the non- US healthcare settings, chlorine was clearly advocated as the disinfectant of choice and it states “*Correctly mixed and applied, chlorine solutions will damage Ebola virus on personal protective equipment (PPE) and other surfaces so that it can no longer infect patients and healthcare workers*”. http://www.cdc.gov/vhf/ebola/hcp/non-us-healthcare-settings.html [6]. In the guidelines for the United States, EPA approved disinfectants are recommended for the disinfection of the environment but there is neither specific mention of the use of chlorine nor of application to PPE and spraying of individuals for personal protection (http://www.cdc.gov/vhf/ebola/healthcare-us/index.html) [16]. In our study, in Sierra Leone, like other EVD affected countries, 0.5 % chlorine was widely used to spray environmental surfaces and humans in contact with suspected or confirmed cases of Ebola, including HCWs while wearing their PPE as they emerged from the Red Zone in ETUs or from community dwellings.

Most accounts of chlorine exposure in humans have been accidental usually following transportation, explosion, inaccurate chlorine mixing, or mechanical malfunction releasing large amounts of chlorine vapour which is inhaled [11–14].

Occupational health authorities including the WHO [17], clearly define chlorine spray exposure as a liquid or gas as being toxic to humans particularly eye, respiratory system and skin [11, 18]. The principal targets of exposure to chlorine gas are the respiratory airways and the eyes. Exposure can occur only by direct contact of inhaled chlorine gas with the respiratory epithelium [19] or via direct contact of the eyes and skin which causes excessive irritation and corneal ulceration [11]; accidental over-chlorination of swimming pools is one type of reported exposure affecting children and adults [11].

A review by D’Alessandro and colleagues [20] reported sensory irritation, and transient pulmonary changes in humans exposed to 1 ppm chlorine causing increased airway resistance and reduced air flow. Yildirim et al. [21] reported morphological alteration in nasal mucosa of rats with loss of cilia and nasal epithelium at concentrations of >5 ppm chlorine.

While our findings are self-reported adverse effects in those who had been exposed to chlorine in the rural and semi urban districts, the number of participants reporting respiratory, skin and eye symptoms particularly amongst healthcare workers who were exposed was significant. This exposure could have been avoided if the use of chlorine had been better regulated and controlled and occupational exposure limits are strictly complied with during production or reconstitution [11, 18]. Anecdotal evidence suggests that the concentrations of chlorine were higher than recommended because of two reasons: often the measurements were inaccurate, and those that were making up the solutions thought a stronger solution would be more effective. If the teams dealing with Ebola were not adequately trained or were unprepared, the excessive use of chlorine was possibly out of fear of contracting the disease [9].

To our knowledge this is the first report of deliberate exposure of humans to chlorine as part of disinfection practices. Our study showed that even a single exposure to 0.5 % chlorine caused ocular, respiratory and skin irritation (Table 3). The frequency of single vs multiple exposure as noted in EVD and HCW groups significantly increased the risk of respiratory and skin irritation particularly in HCWs (Table 4). It is noteworthy, that the use of the available personal protective equipment for HCWs did not have any impact on the adverse effects of chlorine (Table 6) and the use of the currently available PPE is called into question by some colleagues working in the field [22].

Of concern was that participants (21/362 (5.8 %)) said they knew of someone who had been sprayed and transported in a closed non ventilated ambulance to an ETU up to 3 h away and was pronounced dead on arrival without a confirmed diagnosis of Ebola; this raises questions about the number of deaths attributed to Ebola which could have been due to other causes.

Chlorine is a widely used disinfectant and can be effectively used in appropriate circumstances for the correct indications. It should be applied as a wipe rather than a spray, the latter causing aerosolization which can cause respiratory symptoms and irritation of the eyes and the skin.

## Conclusion

Our results point to two main findings; firstly, there was a statistically significant increase in the clinical symptoms of HCW when they were exposed multiple times to chlorine spray. The use of chlorine to disinfect personal protective equipment while HCW are wearing it is not only without evidence, ineffectual but can be hazardous to health [11]. Secondly, the available PPE that was used by HCW did not protect them against the adverse effects of chlorine spray [17]. This was because when chlorine is inhaled it converts to hydrochloric acid in the respiratory tract and causes severe clinical symptoms [19, 20, 23].

We strongly recommend that the spraying of humans with chlorine is banned forthwith; however, appropriate concentration of chlorine may be used for environmental disinfection when recommended by IPC teams. Should chlorine be indicated for disinfection, it should be applied as a wipe rather than a spray. Alternatives such as 70 % alcohol should be considered.
